# Surface Electromyography (sEMG) Activity of Masticatory Muscle (Masseter and Temporalis) with Three Different Types of Orthodontic Bracket

**DOI:** 10.1155/2021/6642254

**Published:** 2021-04-21

**Authors:** Shamima E. Nishi, Norma Ab Rahman, Rehana Basri, Mohammad K. Alam, Nor Farid M. Noor, Siti Aishah Zainal, Adam Husein

**Affiliations:** ^1^Orthodontic Unit, School of Dental Sciences, Health Campus, Universiti Sains Malaysia, 16150 Kubang Kerian, Kelantan, Malaysia; ^2^Hospital Universiti Sains Malaysia, Health Campus, Universiti Sains Malaysia, 16150 Kubang Kerian, Kelantan, Malaysia; ^3^Department of Medicine (Neurology), College of Medicine, Jouf University, Sakaka, Saudi Arabia; ^4^Orthodontic Department, College of Dentistry, Jouf University, Sakaka, Saudi Arabia; ^5^Anatomy Unit, School of Dental Sciences, Health Campus, Universiti Sains Malaysia, 16150 Kubang Kerian, Kelantan, Malaysia; ^6^Basic Sciences and Oral Biology Unit, School of Dental Sciences, Health Campus, Universiti Sains Malaysia, 16150 Kubang Kerian, Kelantan, Malaysia; ^7^Prosthodontic Unit, School of Dental Sciences, Universiti Sains Malaysia, Health Campus, Universiti Sains Malaysia, 16150 Kubang Kerian, Kelantan, Malaysia

## Abstract

**Objective:**

This pre-post study is aimed at determining the effects of masticatory muscle activity (masseter and temporalis) measured via sEMG between conventional, self-ligating, and ceramic bracket after six months of orthodontic treatment.

**Methods:**

A total of eighteen (18) malocclusion patients were identified. Malocclusion patients were subdivided into 3 groups based on the bracket selection (conventional, self-ligating, and ceramic bracket) with 6 patients for each group. sEMG of muscles were done using a two-channel electromyography device, where pregelled and self-adhesive electrodes (bilateral) were applied. Chewing and clenching of masseter and temporalis muscle activity were recorded for 20 s pre and 6 months of orthodontic treatment using sEMG (frequency 60 Hz). The data were analysed by using repeated measures ANOVA in IBM SPSS Statistics Version 24.0.

**Results:**

Chewing and clenching for masseter muscle showed no significant difference (*P* > 0.05) in sEMG activity of three types of the brackets. However, for temporalis muscle, there was a significant difference found in sEMG activity during chewing (*P* < 0.05) and clenching (*P* < 0.05) between these three brackets.

**Conclusion:**

The activity of temporalis muscle showed significant changes in chewing and clenching, where the conventional group demonstrated better muscle activity pre and at six months of fixed appliances.

## 1. Introduction

Electromyography (EMG) is a technique to measure, record, and analyse the myoelectric signals of muscle activities [1]. Surface electromyography (sEMG) can be used to evaluate and monitor the masticatory and facial muscle activity [2]; besides, it can recognize the physiological and pathological circumstances of the stomatognathic system. Aesthetics and function are two parameters which should be considered harmony in all dental procedures [3, 4]. Wide use of the sEMG in clinical and research areas showed that the application of this SEMG is easy and convenient to the user. Data can be recorded from sEMG includes details of activity of the muscles in resting form, chewing, sucking, swallowing, clenching, and contraction of the orofacial muscles [5–8]. This is because different action potentials from different ranges [9] of myofunctional activity or movement can be recorded. Misaligned teeth can affect chewing and clenching [10] through elevation, proclination, and retraction of the masticatory muscle. Determining maxilla-mandibular relationships from EMG activity of masticatory muscle could help in orthodontic field [11].

In the markets, there are a few types of orthodontic brackets depending on the ligation systems [12, 13]. The selections of the brackets are basically based on several factors which include patients' preferences, price, availability, popularity, and visibility [14–16]. However, the effects of fixed appliances on muscle activity are still unknown [17]. This current study is aimed at investigating the effects of masticatory muscle (masseter and temporalis) activity measured via sEMG between three different bracket types (conventional, self-ligating, and ceramic bracket) pre and at six months of fixed appliances.

## 2. Materials and Methods

Prior to the study, all participants provided their verbal and written informed consent. The study was approved by the Human Research Ethics Committee of Universiti Sains Malaysia (USM/JEPeM/15120575).

### 2.1. Subjects

The subjects of this study consisted of 18 malocclusion patients (4 males and 14 females). The subject group was referred to Orthodontic Specialist Clinic, Hospital Universiti Sains Malaysia. Patient with Class II division I malocclusion with a convex profile was included in this study following the inclusion and exclusion criteria. The inclusion criteria involved patients aged from 18 to 30 years old, no history of previous fixed appliances, and all sound teeth (excluding the third molar).

Patients with periodontally compromised, interproximal caries/restoration, temporomandibular joint (TMJ) dysfunction, parafunctional habit, craniofacial deformity, and neuromuscular disease which may interfere the activity of electromyographic have been excluded from this study. Patient with a known case of having sensitivity to the electrode was also excluded in this study.

### 2.2. Sample Size Calculation

PS-Power and Sample Size Calculations software (Version 3.1.2) was used to calculate the sample size based on comparing means. The mean response of muscle activity within each subject group was normally distributed with a standard deviation of 0.22. If the true difference means is 0.4, 6 subjects were needed in each group, a total of 18 subjects were able to reject the null hypothesis that the population means of the groups are equal with probability (power) 0.8. The type I error probability associated with this test of this null hypothesis is 0.05.

### 2.3. Clinical Examination

Intraoral examination was performed to diagnose the malocclusion (according to the British Standard Institute classification of malocclusion). Patients were screened clinically and radiographically.

### 2.4. Study Procedure

Selected patients were randomized using the conventional lottery method with a single blinding method. Eighteen patients were divided into three groups based on the different bracket types (six patients with conventional, six with self-ligating, and six with ceramic brackets). The high-resolution computerized electromyography device sEMG (MyoTrac Infiniti 2 Channel sEMG w/Rehab Suite & Continence Suite-T9855; Quebec, Canada) at 60 Hz and Infiniti software were used to record masticatory muscle (masseter and temporalis) activity for chewing and clenching. These activities were documented two times, prebonding and at six months after fixed appliances.

### 2.5. Electrode Placement

**Figure 1:**
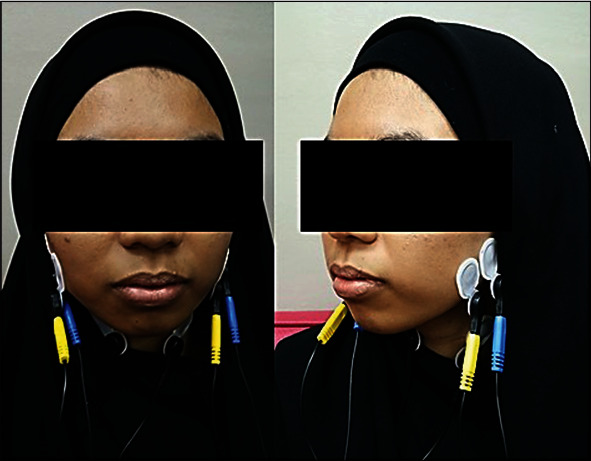
Surface electrode placement over the masseter muscle during sEMG recording.

**Figure 2:**
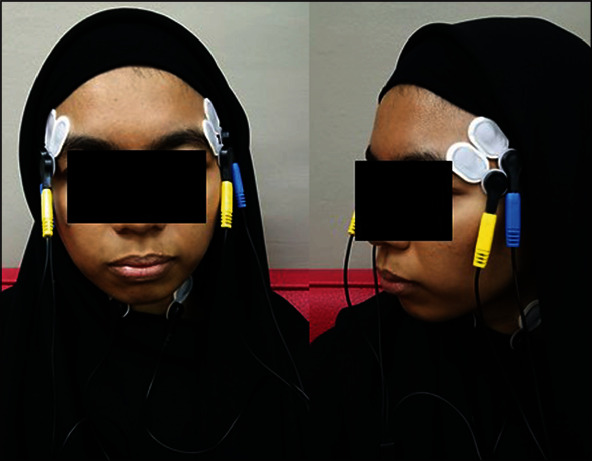
Surface electrode placement over the temporalis muscle during sEMG recording.

Before placing the electrodes over the muscle area, the muscle location was palpated by using fingers. During palpation of the muscles, patients were instructed to clench, and this clenching action helped the researcher to find out the appropriate muscle location by their prominence. Pregelled and self-adhesive electrodes (Ambu Neuroline 720 Electrodes, Denmark) with 3 surface leads (2 recording electrodes, 1 reference) were used. The electrode was placed over the muscle (anterosuperior to the angle of the mandible) on both sides (Figure 1) for recording masseter muscle, while for temporalis muscle, electrodes were placed above a line (drawn from upper earline to canthus of the eye) (Figure 2) with interelectrode distance which was 8 mm.

### 2.6. Patient's Position

Patients in the upright position with eyes opened and no head support and the Frankfort horizontal plan were parallel to the floor. A patient was asked to be seated in a comfortable chair.

### 2.7. Calibrations

Intra- and interexaminer calibration was performed for this study. The masticatory muscle actions (chewing clenching) that are used for calibration were similar to the actions used for data collection. The calibration exercise was performed at the study location. The result has shown that the percentage of agreement was high for all categories and the Kappa score was range from 0.80 to 0.90 showed a moderate to strong agreement between examiners.

### 2.8. sEMG Recording

A chewing gum with 2.7 g was given to patients, and the process of chewing was only started on signal and stopped by a command. Patients were instructed to chew like normal without any interruptions. The process of EMG recording for chewing was at rest for 5 s, followed by 10 s of chewing on the right side and 5 s of rest for both left and right sides. After that, clenching of the right was recorded together at rest for 5 s, 10 s of continuous clenching, and ending with another 5 s of rest. Both muscle activities were recorded following this protocol accordingly.

### 2.9. sEMG Signal Analysis

Mean, median frequency, SD, root mean square (RMS), minimum, maximum, and range of muscle activity were calculated by sEMG software (BioGraph Infiniti, Canada). Microvolts (*μ*V) were used for recorded muscle activity. Before comparing the results of muscle activity between patients, sEMG amplitude values were normalized. In this study, maximum (peak) activation levels during maximum contractions (MVC) were used as a method of normalization. The peak values of every 2.5 seconds were calculated with four repetitions. The mean of these values was calculated for normalizing the muscle activity for each patient.

### 2.10. Statistical Analysis

The IBM SPSS (Statistical Package for the Social Sciences) Version 24.0 was applied, and the confidence level was set at 5% (*P* < 0.05). The mean amplitude for the muscle was compared with three types of brackets. To determine the sEMG activity of muscle between different bracket types, repeated measures ANOVA was performed.

## 3. Results and Discussion

The result showed during chewing, no significant difference was found in the sEMG activity for masseter muscle *F* (7.44, 66.93) = 1.18, *P* = 0.324, and clenching *F*(6.46, 58.15) = 1.69, *P* = 0.134, between three types of the brackets (conventional, self-ligating, and ceramic) (Tables 1 and 2). However, during chewing and clenching, a significant difference *F* (10.26, 92.29) = 2.13, *P* = 0.028, and *F* (9.64, 86.00) = 2.46, *P* = 0.013, respectively, was observed in the sEMG activity of temporalis muscle (Tables 3 and 4).

Aesthetic, function, and dental health are the aim to obtain after orthodontic treatment. However, muscle harmony and balance are often ignored by some clinicians. Abnormal muscle activity can affect the final result which leads to relapse and instability which requires prolong and lifetime of retainers [18, 19] due to a lack of information regarding the effect of the types of the brackets to the muscle activities [20]. Due to the limitation of the research focusing on this aspect, a direct comparison is not possible. This current study was conducted to evaluate the masticatory muscle specifically for masseter and temporalis muscle activity between three different types of brackets. This study found the muscle activity was higher in the conventional bracket group although no significant difference was found for chewing and clenching activity of masseter muscle. Temporalis muscle showed a significant finding for both clenching (*P* = 0.028) and chewing (*P* = 0.013) between three bracket groups. The conventional and ceramic bracket groups showed better temporalis muscle activity compared with the self-ligating bracket for both activities.

Previously, a study conducted by Winocur and colleagues showed a few changes occur in the stomatognathic system [21]. In addition, 24 h following bracket placement, the masticatory performance was significantly reduced [22]. These results were in line with this current study, where we can see a reduction in the chewing efficiency of the masseter and temporalis muscle after six months of treatment with fixed appliances. However, in contrast, no remarkable changes were noted in the maximal clenching activity of the masseter muscle after six months of orthodontic treatment besides discomfort or pain or modification of the occlusal relationship between the maxillary and mandibular dentitions [23].

This current study also found the conventional bracket showed better muscle function compared to self-ligating and ceramic brackets. It indicated that the conventional bracket has altered the activity of the muscle when compared to pre and at six months of treatment. A bigger sample size with a different class of malocclusion can be carried out for future studies to explore the effect of the different brackets to the muscle activities in different malocclusions.

## 4. Conclusion

With the limitation of the current study, patients receiving conventional brackets showed better sEMG activity compared to self-ligating and ceramic bracket groups.

## Figures and Tables

**Table 1:** sEMG activity of masseter muscle chewing between different bracket types.

Masseter muscle evaluation	Mean difference	95% CI for difference		*F* (df)	*P* value
		Lower bound	Upper bound		
Conventional bracket-self-ligating bracket	10.46	-26.32	47.24	1.18 (7.44, 66.93)	0.324
Conventional bracket-ceramic bracket	12.76	-24.02	49.53		
Self-ligating bracket-ceramic bracket	2.29	-34.48	39.07		

CI = confidence interval; df = degree of freedom.

**Table 2:** sEMG activity of masseter muscle clenching between different bracket types.

Masseter muscle evaluation	Mean difference	95% CI for difference		*F* (df)	*P* value
		Lower bound	Upper bound		
Conventional bracket-self-ligating bracket	16.48	-14.29	47.25	1.69 (6.46, 58.15)	0.134
Conventional bracket-ceramic bracket	3.88	-26.89	34.65		
Self-ligating bracket ceramic bracket	-12.60	-43.37	18.17		

CI = confidence interval; df = degree of freedom.

**Table 3:** sEMG activity of temporalis muscle chewing between different bracket types.

Temporalis muscle evaluation	Mean difference	95% CI for difference		*F* (df)	*P* value
		Lower bound	Upper bound		
Conventional bracket-self ligating bracket	11.50	-7.05	30.05	2.13 (10.26, 92.29)	0.028
Conventional bracket-ceramic bracket	0.21	-18.34	18.76		
Self-ligating bracket-ceramic bracket	-11.29	-29.84	7.26		

CI = confidence interval; df = degree of freedom.

**Table 4:** sEMG activity of temporalis muscle clenching between different bracket types.

Temporalis muscle evaluation	Mean difference	95% CI for difference		*F* (df)	*P* value
		Lower bound	Upper bound		
Conventional bracket-self-ligating bracket	23.56	-5.71	52.82	2.46 (9.64, 86.00)	0.013
Conventional bracket-ceramic bracket	1.15	-28.11	30.42		
Self-ligating bracket-ceramic bracket	-22.40	-51.67	6.86		

CI = confidence interval; df = degree of freedom.

## Data Availability

The data underlying this study are included within the article.
